# Resting-state EEG microstate dynamics indicate altered brain network temporal organization in age-related hearing loss

**DOI:** 10.3389/fnagi.2026.1850657

**Published:** 2026-08-03

**Authors:** Yurun Chen, Qiaoyu Liu, Yaohan Chen, Min Zhu, Yufei Qiao, Jiaran Chen, Wen Sun, Hua Yang, Yingying Shang

**Affiliations:** Department of Otorhinolaryngology, Peking Union Medical College Hospital, Chinese Academy of Medical Sciences & Peking Union Medical College, Beijing, China

**Keywords:** age-related hearing loss, brain network, electroencephalogram, microstate, resting-state

## Abstract

**Objective:**

Age-related hearing loss (ARHL) is increasingly recognized as a condition that involves changes in the central nervous system beyond the auditory pathway, with growing evidence showing altered large-scale brain network connectivity. However, how ARHL reshapes the temporal organization of large-scale brain networks remains poorly understood. This study aimed to investigate whether ARHL is associated with alterations in resting-state electroencephalogram (EEG) microstate dynamics and to examine the relationship between microstate features and auditory function.

**Methods:**

Resting-state EEGs were recorded in 45 older adults with ARHL and 50 age-matched normal-hearing (NH) controls. Microstate analysis was performed using a seven-class model. Group differences in microstate parameters were assessed. Correlation analyses were conducted to examine associations between microstate metrics and audiological measures, including pure-tone audiometry (PTA) thresholds and speech-in-noise (SIN) perception.

**Results:**

Compared with NH controls, participants with ARHL exhibited reduced microstate G time coverage at the nominal significance level (uncorrected *p* < 0.05, FDR-adjusted *q* ≥ 0.05). In addition, the ARHL group showed significantly (FDR-adjusted *q* < 0.05) increased transition probabilities (TPs) from C to F and from F to C. Conversely, the TP from D to G was significantly reduced, whereas the TP from G to D showed a reduction at the nominal significance level. Across all participants, after controlling for age and sex, microstate G time coverage and TP from D to G were negatively correlated with PTA thresholds, whereas the TPs from C to F and from F to C were positively correlated with PTA thresholds. Furthermore, microstate G time coverage showed a nominal negative correlation with the SIN threshold.

**Conclusion:**

ARHL is associated with altered resting-state EEG microstate dynamics, mainly reflected by changes in TPs among sensorimotor and higher-order cognitive networks. Resting-state EEG microstate analysis provides a comprehensive and systematic framework for studying brain network temporal reorganization induced by auditory decline.

## 1 Introduction

Age-related hearing loss (ARHL), also referred to as presbycusis, is among the most prevalent sensory impairments in older adults (Agrawal et al., 2009). Epidemiological data indicate that more than one-third of individuals older than 65 years and more than half of those older than 75 years exhibit clinically significant hearing decline (World Health Organization, 2021). ARHL typically presents as a bilateral, progressive, high-frequency sensorineural deficit (Gates and Mills, 2005; Bowl and Dawson, 2019). Its pathological basis includes degeneration of cochlear hair cells, loss of spiral ganglion neurons, atrophy of the stria vascularis, and disruption of synapses between inner hair cells and auditory nerve fibers (Kujawa and Liberman, 2015; Schuknecht and Gacek, 1993). Current clinical management focuses primarily on peripheral rehabilitation through hearing aids or cochlear implantation (Haensel et al., 2005; Migirov et al., 2010; Orabi et al., 2006). However, outcomes vary substantially across individuals. Many patients continue to report difficulties, particularly in understanding speech in noisy environments, despite appropriate amplification (Peelle and Wingfield, 2016; Recanzone, 2018). These clinical observations suggest that ARHL extends beyond peripheral dysfunction and involves alterations in central neural function.

Accumulating evidence links ARHL to structural and functional changes within the central nervous system. Previous studies have demonstrated associations between hearing loss and accelerated cognitive decline and between hearing loss and increased risk of dementia (Lin et al., 2011; Livingston et al., 2020). Neuroimaging of individuals with ARHL has further revealed altered connectivity within large-scale networks such as the default mode network (DMN), dorsal attention network (DAN), salience network and sensorimotor-related systems (Schulte et al., 2020; Husain et al., 2014). Such findings suggest that ARHL may affect not only auditory pathways, but also broader systems involved in attention, sensory integration and cognitive control. Cortical reorganization in ARHL is not limited to severe hearing impairment. Even mild-to-moderate ARHL has been associated with cross-modal plasticity. In this process, visual and somatosensory systems may recruit auditory cortical regions after reduced auditory input (Sharma and Glick, 2016; Glick and Sharma, 2017). This redistribution of neural resources may support compensation during degraded listening.

Electroencephalography (EEG) provides millisecond-level temporal resolution, enabling a non-invasive investigation of large-scale brain dynamics (Lehmann, 1984). Microstate analysis is a well-established approach in which the continuous EEG signal is segmented into a series of temporally discrete and nonoverlapping scalp potential maps (Tarailis et al., 2024). These representative topographies are then reassigned to the ongoing signal using a competitive back-fitting procedure based on spatial correlation (Khanna et al., 2015; Koenig et al., 2002). Microstates are considered electrophysiological correlates of large-scale functional networks. Recent microstate studies have increasingly used seven-state solutions, as they provide a more fine-grained description of resting-state brain dynamics than the classical four-state model (Custo et al., 2017; Tarailis et al., 2021; Tarailis et al., 2024). Previous EEG-fMRI, source-localization, and review studies have linked microstate classes to partly distinct neural systems (Britz et al., 2010; Custo et al., 2017; Tarailis et al., 2024). Microstate A has been associated primarily with auditory processing and arousal-related activity. Microstate B has been linked to visual processing and self-related visual imagery. Microstate C has been associated with default-mode-related activity, especially posterior DMN regions. Microstate D has been linked to attention, executive control, and externally oriented processing. Microstate E has been associated with salience-related, interoceptive, and emotional processing. Microstate F has been linked to anterior DMN-related and anterior cingulo-frontal/insular dynamics. Microstate G is less well characterized, but it has been associated with sensorimotor dynamics. This seven-class framework may therefore provide a useful approach for examining the temporal organization of large-scale brain states.

Microstate analysis has been applied to auditory-related disorders, particularly tinnitus and sudden sensorineural hearing loss. Previous studies have reported altered microstate duration and transition dynamics in these conditions, suggesting central reorganization after auditory dysfunction (Cai et al., 2019; Cai et al., 2018). Additional work has linked microstate transition probabilities (TPs) to tinnitus severity, supporting the potential of microstate metrics as markers of altered brain dynamics (Wang et al., 2024). These findings suggest that auditory dysfunction can be accompanied by measurable changes in the temporal organization of large-scale brain states.

Despite these advances, resting-state EEG microstate alterations in ARHL have not been systematically examined using an extended seven-class microstate framework. Unlike acute auditory disorders, ARHL is characterized by a gradual, long-term sensory decline. This process occurs together with aging-related neural changes. Investigating microstate dynamics in ARHL may help clarify how chronic auditory degradation is related to large-scale brain-state organization.

The present study compared resting-state EEG microstates in older adults with ARHL with age-matched individuals with normal hearing (NH). We further examined the associations between microstate dynamics and audiological measures, including pure-tone audiometry (PTA) thresholds and speech-in-noise (SIN) thresholds. This work aimed to determine whether ARHL is associated with altered microstate parameters and transition patterns. It also aimed to explore whether these EEG microstate features are related to hearing severity and SIN perception.

## 2 Methods

### 2.1 Participants

**Figure 1:**
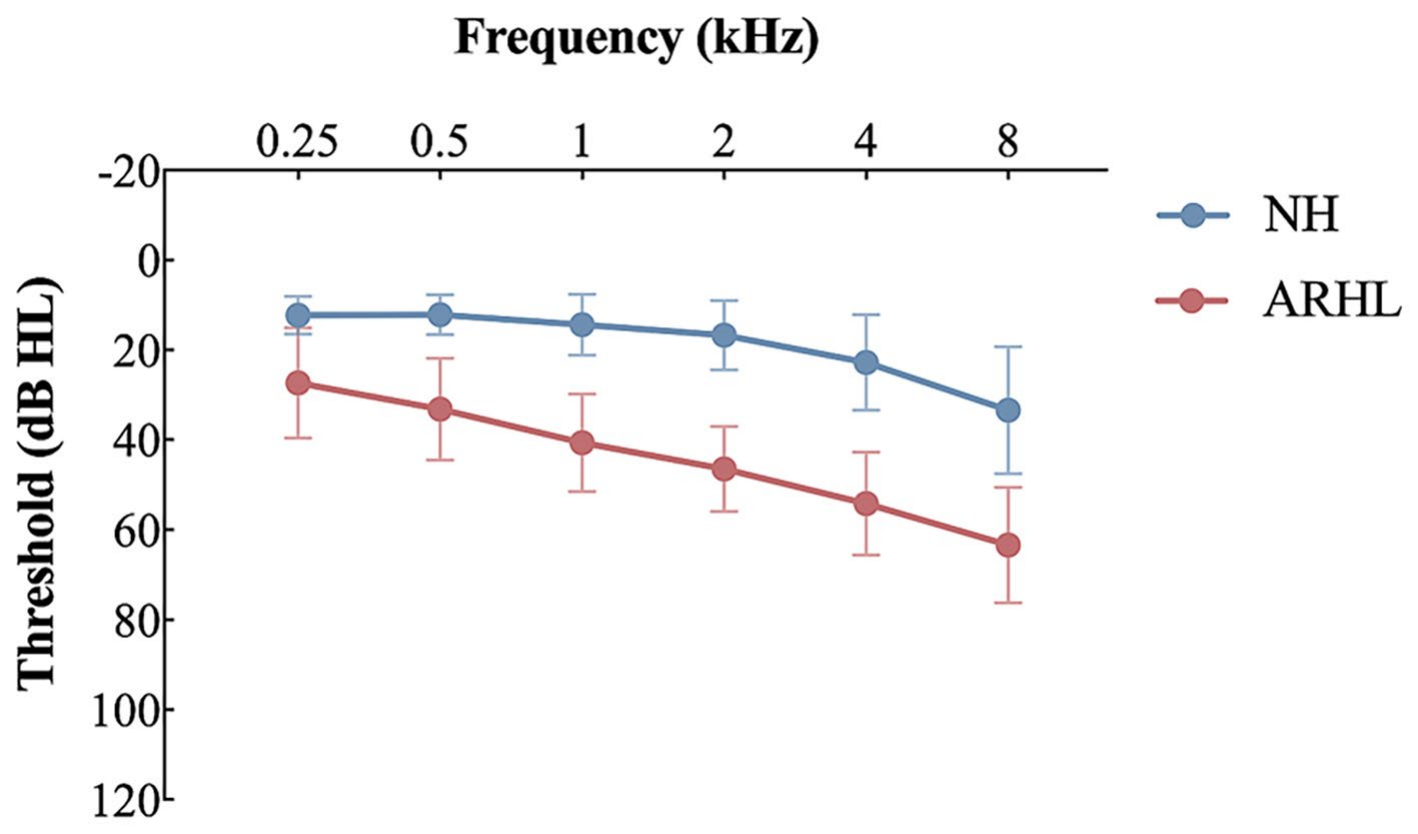
Average PTA thresholds for participants in the NH and ARHL groups. PTA, pure-tone audiometry; ARHL, age-related hearing loss; NH, normal hearing.

Forty-five participants with ARHL (20 males; age 67.16 ± 3.46 years, range 61–73 years) were recruited from the Department of Otorhinolaryngology at Peking Union Medical College Hospital and retired staff as well as their families at Peking Union Medical College. The ARHL group included individuals with mild-to-moderate symmetric sensorineural hearing loss (Figure 1), defined as (1) an air–bone gap ≤10 dB; (2) mean air conduction thresholds at 0.5–4 kHz ≥25 dB HL and <60 dB HL; and (3) an interaural threshold difference ≤15 dB HL across all frequencies. None had a history of hearing aid use. Fifty NH controls (24 males; age 66.38 ± 3.19 years, range 60–74 years) were recruited from retired staff and their families at Peking Union Medical College. The two groups were matched for age, sex, education level, and handedness (Table 1). Participants with possible cognitive impairment were excluded on the basis of the Montreal Cognitive Assessment (MoCA < 26). All participants were in good general health, had normal or corrected-to-normal vision, and had no history of ear disease (e.g., otitis media, cholesteatoma, or conductive hearing loss), neurological or psychiatric disorders, or major head trauma. Participants whose EEGs were of poor quality were also excluded. Data on vascular risk factors, including hypertension, hyperglycemia, hyperlipidemia, smoking, and alcohol use, were collected. No significant between-group differences were detected. The study was approved by the Ethics Committee of Peking Union Medical College Hospital. All participants provided written informed consent.

**Table 1:** Demographic data, auditory ability, and risk factors for cerebrovascular diseases in the two groups.

Variable	NH (*n* = 50)	ARHL (*n* = 45)	Test statistic	*p*-value
Age [year, mean (SD)]	66.38 (3.19)	67.16 (3.46)	*t =* −1.135	*p =* 0.259
Male [*n* (%)]	24 (48.0)	20 (44.4)	*χ^2^ =* 0.120	*p* = 0.729
PTA threshold [dB HL, mean (SD)]	16.35 (4.60)	43.57 (8.08)	*t =* −19.895	***p* < 0.001** ^*^
SIN threshold [dB, mean (SD)]	−1.31 (1.13)	0.93 (1.98)	*t =* −6.601	***p* < 0.001** ^*^
MoCA [mean (SD)]	27.94 (1.24)	28.00 (1.22)	*t =* −0.236	*p =* 0.814
Education time [year, mean (SD)]	12.84 (2.48)	12.58 (3.15)	*t =* 0.445	*p =* 0.658
Right-handed [*n* (%)]	49 (98.0)	41 (91.1)	*χ^2^ =* 2.254	*p* = 0.133
Hypertension [*n* (%)]	11 (22.0)	11 (24.4)	*χ^2^ =* 0.080	*p* = 0.778
Hyperglycemia [*n* (%)]	4 (8.0)	4 (8.9)	*χ^2^ =* 0.024	*p* = 0.876
Hyperlipidemia [*n* (%)]	13 (26.0)	15 (33.3)	*χ^2^ =* 0.613	*p* = 0.434
Smoking [*n* (%)]	8 (16.0)	6 (13.3)	*χ^2^ =* 0.134	*p* = 0.714
Alcohol use [*n* (%)]	6 (12.0)	4 (8.9)	*χ^2^ =* 0.243	*p* = 0.622

NH, normal hearing; ARHL, age-related hearing loss; PTA, pure-tone audiometry; SIN, speech-in-noise test; MoCA, Montreal Cognitive Assessment; SD, standard deviation. Bold values indicate *p* < 0.05; ^*^p < 0.05.

### 2.2 Audiological tests

In the present study, PTA, which primarily reflects peripheral auditory function, was used to assess hearing acuity. In addition, SIN perception was evaluated as a measure of central auditory function.

#### 2.2.1 PTA

PTA was performed in a soundproof booth in accordance with ISO 8253-1:2010. The mean hearing threshold was defined as the average of the thresholds at 0.5, 1, 2, and 4 kHz. The average PTA threshold of both ears was recorded for each participant.

#### 2.2.2 SIN perception

The SIN assessment was conducted via the Hearing-in-Noise Test (HINT; Bio-Logic Systems Corp., Mundelein, IL, United States) using Mandarin HINT sentences in a soundproof booth (Soli and Wong, 2008). Mandarin sentences, together with speech-shaped noise, were presented through a loudspeaker that was positioned 1 meter away from and directly in front of the participants. The intensity level of the sentences was fixed at 65 dB SPL, and the volume of the noise was adjusted according to the individual’s reaction. The SIN threshold was defined as the signal-to-noise ratio at which the participant achieved 50% correct sentence repetition.

### 2.3 EEG recording

EEG data were recorded using 64 Ag–AgCl scalp electrodes arranged according to the extended 10–20 system (Neuroscan SynAmps2 8050; Compumedics, Australia) and collected with CURRY 8 software. The signals were amplified with a gain of 2010 and bandpass-filtered from 0.01 to 100 Hz. The data were digitized at a sampling rate of 1,000 Hz. Electrode impedance was maintained below 10 kΩ throughout the recording. For each participant, a resting-state EEG was recorded for 4 min with the participant’s eyes closed.

### 2.4 EEG data preprocessing

The EEG data were preprocessed using EEGLAB (version 2026.0.0), an open-source toolbox running in the MATLAB (version R2024a, 24.1.0.2537033) environment (MathWorks Inc., Natick, MA, United States) with an automated quality-control pipeline. Raw EEG data were imported and band-pass filtered from 1 to 40 Hz, followed by downsampling to 250 Hz when necessary. Continuous high-amplitude artifacts were removed using artifact subspace reconstruction implemented in the clean_rawdata EEGLAB plugin, with a BurstCriterion of 20 and a WindowCriterion of 0.25. Bad channels were detected before independent component analysis (ICA) using clean_rawdata criteria, including a flatline criterion of 5 s, a channel correlation criterion of 0.80, and a line-noise criterion of 4 standard deviations. Bad channels were removed before ICA and restored after ICA-based artifact correction using spherical interpolation. ICA was performed on the cleaned continuous data using the extended Infomax algorithm implemented in EEGLAB’s runica function. ICA was performed with PCA dimensionality reduction based on the effective data rank. Components were automatically classified using ICLabel. Components were removed if they were classified as eye components with probability ≥0.90 and brain probability <0.20, muscle components with probability ≥0.80 and brain probability <0.20, or heart components with probability ≥0.80 and brain probability <0.20. After artifact component removal, interpolated data were re-referenced to the average reference.

Because the resting-state EEG data were processed as continuous data, no fixed-length epochs were generated during preprocessing; therefore, the number of retained epochs was not applicable. Instead, the amount of retained continuous data after ASR was recorded. In the final EEG dataset, 95 participants were included. On average, 88.91 ± 12.58% of the continuous data was retained after ASR, with a range of 51.51–100.00%. The average final data length was 197.05 ± 30.47 s. An average of 3.13 ± 2.76 channels were interpolated per participant, and 1.34 ± 1.72 independent components were removed per participant.

### 2.5 EEG microstate analysis

Microstate analysis was performed using the MicrostateLab plugin implemented in EEGLAB. The global field power (GFP) of the preprocessed EEG signal was first calculated, reflecting the spatial standard deviation of scalp potentials across electrodes at each time point;


$$\mathrm{GFP}(t)=\sqrt{\frac{1}{K}\sum_{i=1}^{K}{\left(V_{i}(t)-\bar{V}(t)\right)}^{2}}$$

where $V_{i}(t)\;$ denotes the potential at electrode i and time t , $\bar{V}(t)\;$ is the mean potential across all electrodes, and K is the total number of electrodes. EEG topographic maps at local maxima of the GFP were extracted to improve the signal-to-noise ratio and reduce temporal redundancy.

The GFP-peak maps were subjected to modified k-means clustering to identify representative microstate classes. Polarity was ignored during clustering, such that maps with opposite polarities but similar topographies were treated as equivalent. The number of microstate classes was set to seven to allow comparison with the seven-class templates summarized by Tarailis et al. (2024) and previous extended microstate literature. Clustering was restricted to GFP peaks, and the maximum number of maps was set to infinity; therefore, no subsampling was applied and all identified GFP peaks were used for clustering. The EEG maps were normalized before clustering, and the k-means procedure was repeated 20 times with different random initializations.

To derive representative group-level microstate templates, clustering was first conducted separately within each group. The NH and ARHL group-level maps were then combined using a weighted averaging approach to generate a common grand-mean template. The grand-mean maps were sorted and labeled according to the seven-microstate template summarized by Tarailis et al. (2024). The resulting grand-mean template was subsequently used to sort the group-level and individual microstate maps, ensuring consistent labeling of microstate classes across participants.

After sorting, topographical outlier detection of the individual microstate maps was performed using the Mahalanobis-distance-based outlier detection procedure implemented in MicrostateLab. Datasets identified as topographical outliers at the predefined threshold of *p* < 0.05 were excluded from further analyses. After exclusion of outlier datasets, the mean and grand-mean microstate maps were regenerated, sorted again, and the outlier detection procedure was repeated until no further topographical outliers were detected.

To evaluate whether the group-level maps could be appropriately combined, the independently derived NH and ARHL group-level maps were compared after sorting with the common seven-class reference template. Topographical similarity was quantified using polarity-invariant shared variance, which corresponds to the squared spatial correlation between maps.

The final grand-mean microstate maps were then used as templates for back-fitting to the cleaned EEG data of each participant. Back-fitting was performed at GFP peaks. No additional minimum segment-duration criterion or temporal smoothing was applied to reject short microstate segments. For each microstate class, a set of temporal and spatial parameters, including global explained variance (GEV), time coverage, mean duration and occurrence rate, was calculated. TPs between microstate classes were also calculated on the basis of the original transition matrix. These parameters were used in subsequent statistical analyses.

The GEV was calculated as the variance explained by each microstate, weighted by the GFP across time (Murray et al., 2008). Time coverage represents the proportion of total recording time occupied by each microstate and reflects the relative temporal contribution of each microstate (Khanna et al., 2015; Murray et al., 2008). The mean duration was defined as the average amount of time during which consecutive EEG topographies remained assigned to the same microstate class, reflecting the synchronized activity of the underlying cortical generators during each occurrence of a given microstate (Khanna et al., 2015). The occurrence rate refers to the average number of times a given microstate appeared per second; it reflects the tendency of intracortical sources to exhibit synchronized activation (Khanna et al., 2015). Microstate TP was defined as the relative frequency of transitions from one microstate to another, normalized by the total number of transitions; it reflects the temporal organization of microstate sequences (Lehmann et al., 2005).

### 2.6 Statistics

Chi-square tests were used to compare the two groups with respect to sex, the prevalence of hypertension, hyperglycemia, hyperlipidemia, history of smoking and drinking, and handedness. Independent-samples t tests were used to compare age, MoCA score, years of education, mean bilateral PTA, and SIN thresholds between the groups. Independent-samples *t*-tests were also applied to examine group differences in EEG microstate parameters. For exported EEG microstate parameters used in group-level statistical analyses, statistical outliers were defined as the group mean by more than three standard deviations and were excluded before the corresponding statistical analyses. Multiple comparisons were corrected using the false discovery rate (FDR) procedure. The significance level was set at 0.05. Cohen’s *d* was calculated to estimate the effect size. Partial correlation analysis with age and sex as covariates was used to assess associations between EEG microstate features and audiological measures. Statistical analyses were conducted using IBM SPSS Statistics version 31.0.0.0.

## 3 Results

### 3.1 Demographic characteristics and auditory function

The demographic characteristics of the subjects are shown in Table 1. There was no significant difference between the NH controls and the individuals with ARHL in age, sex, MoCA score, years of education, handedness, hypertension status, hyperglycemia status, hyperlipidemic status, smoking status, or drinking history. Table 1 also shows the audiological results. Compared with the NH controls, the individuals with ARHL had significantly higher PTA thresholds (*t* = −19.895, d*f* = 93, *p* < 0.001) and SIN thresholds (*t* = −6.601, d*f* = 92, *p* < 0.001).

### 3.2 Comparison of microstate metrics between groups

**Figure 2:**
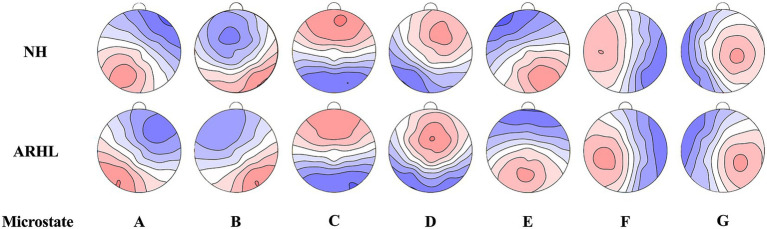
Topographical maps showing the clustering of microstates for the NH and ARHL groups. ARHL, age-related hearing loss; NH, normal hearing.

Seven canonical microstates (A–G) were identified across all the participants. The mean microstate maps obtained from both the ARHL and the NH groups are shown in Figure 2. The shared variances between corresponding NH and ARHL group-level maps ranged from 82.0 to 96.2%, corresponding to polarity-invariant spatial correlations of approximately 0.906 to 0.981. The grand-mean microstate maps showed substantial topographical similarity to the established seven-class template summarized by Tarailis et al. (2024), with shared variances between corresponding maps ranging from 79.9 to 96.1%, corresponding to polarity-invariant spatial correlations of approximately 0.894 to 0.980. This supports the comparability of the present microstate labels with previously reported seven-class microstate frameworks. Statistical analysis revealed no significant group differences in GEV, mean duration, or occurrence rate across microstates, suggesting that these conventional microstate parameters were broadly comparable between the two groups (Table 2).

**Table 2:** Comparison of the microstate parameters between the two groups.

Parameter	Microstate	NH (*n* = 50)	ARHL (*n* = 45)	*t*	*p*-value	Cohen’s *d*
GEV [mean (SD)]	A	0.137 (0.047)	0.130 (0.054)	*t =* 0.617	*p =* 0.539	*d =* 0.127
	B	0.103 (0.033)	0.113 (0.041)	*t =* −1.387	*p =* 0.169	*d =* −0.285
	C	0.158 (0.087)	0.174 (0.083)	*t =* −0.952	*p =* 0.344	*d =* −0.196
	D	0.097 (0.062)	0.085 (0.055)	*t =* 1.005	*p =* 0.318	*d =* 0.208
	E	0.073 (0.031)	0.081 (0.045)	*t =* −1.089	*p =* 0.279	*d =* −0.226
	F	0.063 (0.035)	0.061 (0.030)	*t =* 0.349	*p =* 0.728	*d =* 0.072
	G	0.051 (0.025)	0.042 (0.025)	*t =* 1.737	*p =* 0.086	*d =* 0.357
	Total	0.691 (0.074)	0.704 (0.056)	*t =* −0.963	*p =* 0.338	*d =* −0.198
Time coverage [%, mean (SD)]	A	18.384 (4.554)	17.683 (5.378)	*t =* 0.684	*p =* 0.496	*d =* 0.141
	B	15.922 (3.389)	17.007 (4.795)	*t =* −1.283	*p =* 0.203	*d =* −0.264
	C	15.109 (5.860)	17.008 (6.523)	*t =* −1.487	*p =* 0.140	*d =* −0.307
	D	14.920 (6.528)	13.757 (7.671)	*t =* 0.798	*p =* 0.427	*d =* 0.164
	E	12.521 (3.961)	12.948 (4.741)	*t =* −0.475	*p =* 0.636	*d =* −0.098
	F	11.750 (4.571)	11.672 (3.914)	*t =* 0.088	*p =* 0.930	*d =* 0.018
	G	10.216 (3.358)	8.686 (3.697)	*t =* 2.114	***p =* 0.037** ^**†**^	*d =* 0.434
Duration [s, mean (SD)]	A	0.039 (0.008)	0.038 (0.007)	*t =* 0.951	*p =* 0.344	*d =* 0.197
	B	0.036 (0.006)	0.037 (0.006)	*t =* −0.505	*p =* 0.615	*d =* −0.104
	C	0.039 (0.010)	0.039 (0.010)	*t =* −0.440	*p =* 0.661	*d =* −0.091
	D	0.035 (0.007)	0.033 (0.008)	*t =* 0.818	*p =* 0.415	*d =* 0.170
	E	0.032 (0.006)	0.032 (0.005)	*t =* 0.224	*p =* 0.823	*d =* 0.046
	F	0.031 (0.005)	0.031 (0.005)	*t =* 0.011	*p =* 0.991	*d =* 0.002
	G	0.029 (0.004)	0.028 (0.005)	*t =* 0.794	*p =* 0.429	*d =* 0.163
Occurrence [times/s, mean (SD)]	A	4.805 (1.175)	4.661 (1.273)	*t =* 0.572	*p =* 0.569	*d =* 0.118
	B	4.516 (0.970)	4.751 (1.409)	*t =* −0.952	*p =* 0.344	*d =* −0.196
	C	3.913 (0.887)	4.324 (1.236)	*t =* −1.873	*p =* 0.064	*d =* −0.385
	D	4.194 (1.546)	3.841 (1.757)	*t =* 1.039	*p =* 0.301	*d =* 0.214
	E	3.920 (1.242)	4.031 (1.317)	*t =* −0.422	*p =* 0.674	*d =* −0.087
	F	3.788 (1.392)	3.906 (1.392)	*t =* −0.410	*p =* 0.683	*d =* −0.085
	G	3.542 (1.254)	3.035 (1.291)	*t =* 1.943	*p =* 0.055	*d =* 0.399

NH, normal hearing; ARHL, age-related hearing loss; GEV, global explained variance; SD, standard deviation; Bold values indicate uncorrected *p* < 0.05; ^**†**^, uncorrected *p* < 0.05, FDR-adjusted *q* ≥ 0.05; *d*, Cohen’s *d* effect size; Positive *d*-values indicate higher values in NH controls; negative *d*-values indicate higher values in individuals with ARHL.

**Figure 3:**
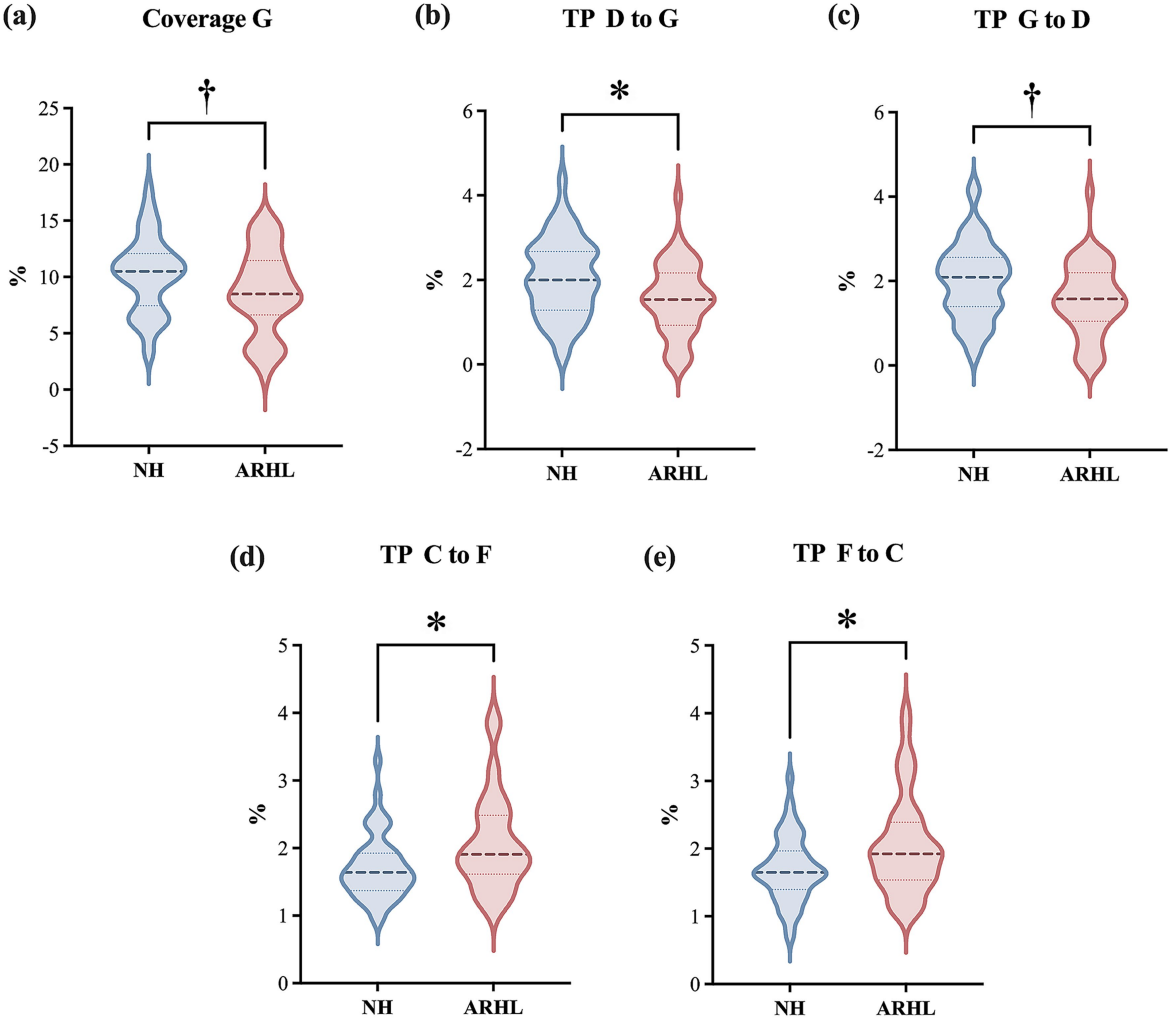
Microstate parameters in the NH and ARHL groups. **(a)** Coverage G in the NH and ARHL groups. **(b)** TP D to G in the NH and ARHL groups. **(c)** TP G to D in the NH and ARHL groups. **(d)** TP C to F in the NH and ARHL groups. **(e)** TP F to C in the NH and ARHL groups. ARHL, age-related hearing loss; NH, normal hearing; TP, transition probability; ^*^, FDR-adjusted *q* < 0.05; ^**†**^, uncorrected *p* < 0.05, FDR-adjusted *q* ≥ 0.05.

In contrast, a nominal between-group difference in time coverage was observed for microstate G. Compared with NH controls, the individuals with ARHL showed lower time coverage of microstate G (coverage G, *t* = 2.114, d*f* = 93, uncorrected *p* = 0.037, *d =* 0.434), indicating that microstate G occupied a smaller portion of the total EEG time in the ARHL group (Table 2; Figure 3). However, this difference did not survive FDR correction.

The TPs for each pair of the seven microstates in the two groups were also compared. Compared with NH controls, individuals with ARHL showed a significantly reduced TP D to G (*t* = 2.732, d*f* = 93, FDR-adjusted *q* = 0.048, *d* = 0.561) (Figure 3). The TP G to D (*t* = 2.445, d*f* = 93, uncorrected *p* = 0.016, *d* = 0.502) was also lower in the ARHL group, though did not survive FDR correction. In contrast, individuals with ARHL showed significantly increased C-to-F (*t* = −2.924, d*f* = 92, FDR-adjusted *q* = 0.024, *d* = −0.604) and F-to-C TP (*t* = −2.821, d*f* = 92, FDR-adjusted *q* = 0.036, *d* = −0.582) compared with NH controls (Figure 3).

### 3.3 Correlation analyses

To further explore the link between EEG microstate findings and audiological function, we examined the associations between audiological measures and microstate parameters showing alterations in the above analyses across all participants. Since ARHL represents a gradual process and the boundary between normal and abnormal hearing loss is clinically defined, these analyses were performed across the entire sample rather than separately within each group.

**Figure 4:**
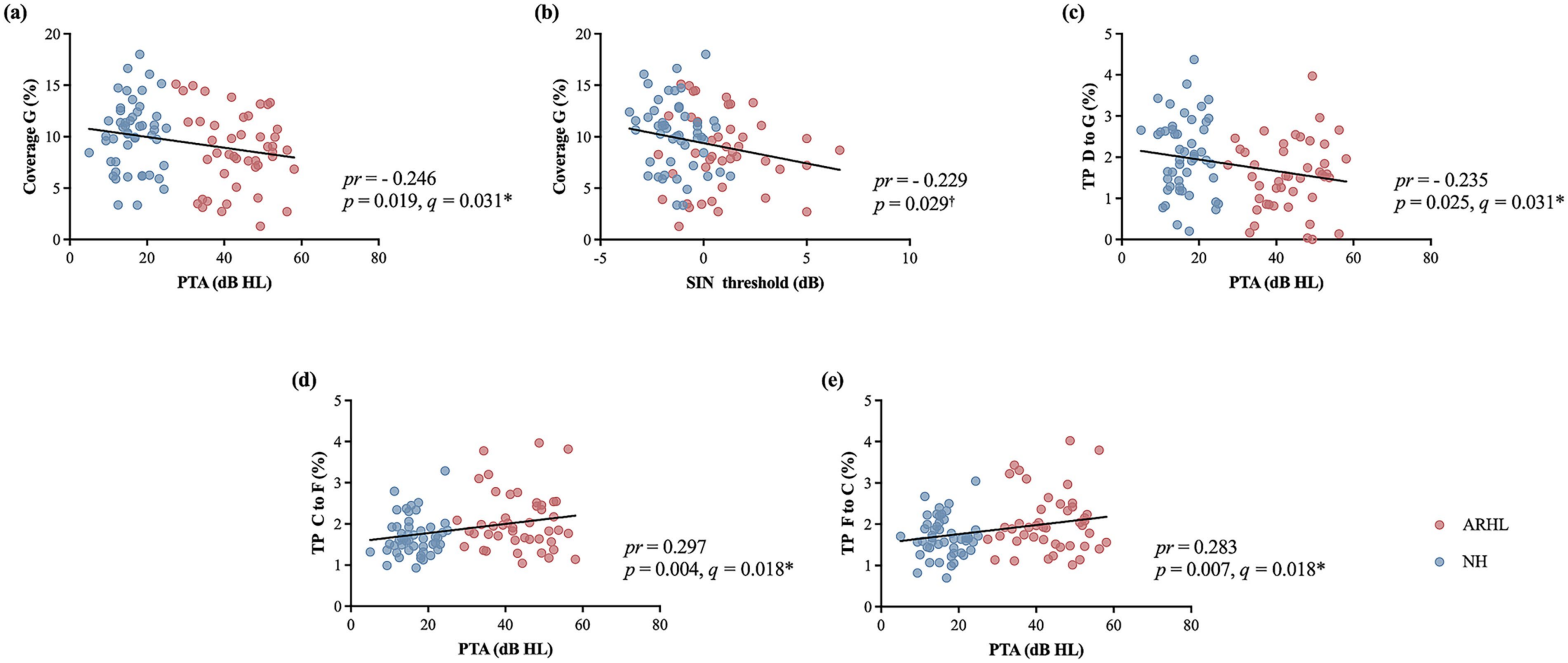
Analysis of correlations between auditory function and microstate parameters in all individuals after controlling for the effects of sex and age. **(a)** Correlations between PTA thresholds and coverage G. **(b)** Correlations between SIN thresholds and coverage G. **(c)** Correlations between PTA thresholds and TP D to G. **(d)** Correlations between PTA thresholds and TP C to F. **(e)** Correlations between PTA thresholds and TP F to C. ARHL, age-related hearing loss; NH, normal hearing; PTA, pure-tone audiometry; SIN, Speech-in-noise test; TP, transition probability. ^*^, FDR-adjusted *q* < 0.05; ^**†**^, uncorrected *p* < 0.05, FDR-adjusted *q* ≥ 0.05.

After controlling for age and sex, coverage G was negatively correlated with PTA threshold (*pr =* −0.246, FDR-adjusted *q =* 0.031) and was also nominally negatively correlated with SIN threshold (*pr =* −0.229, uncorrected *p* = 0.029) (Figure 4). In addition, TP D to G was negatively correlated with PTA threshold (*pr* = −0.235, FDR-adjusted *q* = 0.031), whereas TP C to F and TP F to C were positively correlated with PTA threshold (TP C to F: *pr* = 0.297, FDR-adjusted *q* = 0.018; TP F to C: *pr* = 0.283, FDR-adjusted *q* = 0.018) (Figure 4).

Furthermore, we explored whether high-frequency hearing loss was associated with the target microstate parameters. High-frequency PTA threshold was defined as the average hearing threshold at 4 and 8 kHz. After controlling for age and sex, high-frequency PTA threshold was negatively correlated with microstate G coverage (*pr* = −0.232, FDR-adjusted *q* = 0.026), TP D to G (*pr* = −0.291, FDR-adjusted *q* = 0.008), and TP G to D (*pr* = −0.269, FDR-adjusted *q* = 0.011). In contrast, high-frequency PTA was positively correlated with TP C to F (*pr* = 0.315, FDR-adjusted *q* = 0.005) and TP F to C (*pr* = 0.317, FDR-adjusted *q* = 0.005).

## 4 Discussion

In this study, we systematically investigated alterations in resting-state EEG microstates in individuals with ARHL compared with their NH peers using an extended seven-class model. To our knowledge, this is the first study to examine ARHL-related microstate alterations within a seven-class microstate framework. The main group differences were found in TPs. This pattern suggests that ARHL may mainly affect the temporal organization of microstate sequences.

Specifically, the ARHL group showed increased microstate C-to-F and F-to-C transitions. It also showed reduced microstate D-to-G transitions. In addition, microstate G coverage and G-to-D transitions were lower at the nominal significance level. These findings suggest altered temporal organization of G-related and C-F-related microstate dynamics in ARHL. Still, they should be interpreted with caution. TP describes the tendency of one microstate to follow another. It does not show causal flow. It also does not prove a fixed cognitive sequence.

Microstate D has been linked to attention, executive control, and externally oriented processing (Britz et al., 2010; Custo et al., 2017; Bréchet et al., 2019; Kim et al., 2021; Tarailis et al., 2024). This interpretation is supported by source-localization and task-based microstate studies. Microstate G is less well characterized. However, seven-class microstate studies have linked G to right inferior parietal, superior temporal, and cerebellar regions (Custo et al., 2017; Tarailis et al., 2021; Tarailis et al., 2024). These areas suggest a possible role in sensorimotor dynamics.

In the present study, ARHL was associated with lower G coverage and reduced D-to-G and G-to-D transitions. These microstate G-related findings may be relevant to sensory and attentional reorganization in ARHL. Higher PTA thresholds were also associated with lower G coverage and lower TP D-to-G. This pattern suggests that more severe hearing loss may be accompanied by weaker G-related engagement and weaker temporal coupling from attention-related to sensorimotor dynamics. The association between lower G coverage and poorer SIN performance further supports a possible link between G-related dynamics and higher-order auditory perception.

This interpretation fits well with previous ARHL research. Resting-state fMRI evidence showed that increasing hearing loss is associated with reduced coupling between the DAN and sensorimotor or motor regions (Schulte et al., 2020). These regions included the precentral and postcentral gyri, and the cerebellum. Other EEG and EEG-fNIRS studies also support altered frontal, parietal, and sensorimotor network involvement in ARHL (Huang et al., 2023; Liu et al., 2025; Wang et al., 2025).

These findings may also be considered in a broader cross-modal framework. Hearing loss can induce cortical reorganization beyond the auditory cortex. Visual and sensorimotor systems may recruit auditory regions after auditory deprivation (Sharma and Glick, 2016; Glick and Sharma, 2017). Frontal regions may also be recruited during effortful listening. Therefore, the present microstate G-related and D-G findings may reflect altered sensorimotor temporal dynamics under chronically degraded auditory input.

The increased bidirectional microstate C-F transitions in ARHL individuals suggest a second form of altered temporal organization. Microstate C has been associated with posterior DMN regions (Custo et al., 2017; Bréchet et al., 2019). These include the posterior cingulate cortex, precuneus, and angular gyrus. Microstate F has also been established to be related to DMN (Custo et al., 2017; Tarailis et al., 2021). However, microstate F should not be treated as a simple extension of microstate C. Previous seven-class studies have linked F to dorsal anterior cingulate, frontal, and insular regions. Thus, F may involve anterior cingulo-frontal or insular dynamics.

In the present study, ARHL exhibited increased microstate C-to-F and F-to-C transitions, indicating enhanced temporal coupling between posterior default-mode-related dynamics and anterior cingulo-frontal/insular dynamics. Furthermore, higher PTA was also associated with higher C-to-F and F-to-C TPs. This pattern suggests that more severe hearing loss may be accompanied by stronger temporal coupling. This does not necessarily mean that DMN activity was globally increased. Rather, it may reflect altered switching between partially separable posterior and anterior network states. This point is important because microstate C and F can be spatially similar and may be merged into lower-class microstate solutions (Custo et al., 2017; Tarailis et al., 2021). A seven-class model may therefore reveal microstate C-F temporal coupling that would otherwise be hidden.

These present microstate C-F findings may be relevant to the neural consequences of degraded hearing. ARHL increases listening effort and may require greater use of domain-general cognitive systems (Peelle and Wingfield, 2016). Prior ARHL studies have reported altered activity or connectivity in frontal, prefrontal, parietal, and default-mode-related regions (Huang et al., 2023; Wang et al., 2025). The present microstate C-F result may therefore be compatible with altered posterior–anterior network coupling in response to chronically degraded auditory input.

The group differences and correlation results suggest a severity-related pattern of altered microstate dynamics in ARHL. More severe hearing loss was linked to weaker microstate G-related engagement and D-G coupling, and to stronger C-F coupling. These findings are consistent with previous evidence of severity-dependent network changes in ARHL. Schulte et al. (2020) found that hearing thresholds were related to reduced resting-state coupling among attention, visual, motor, and sensorimotor networks. Other studies also showed that hearing loss can affect central auditory processing and cortical network organization (Recanzone, 2018; Peelle and Wingfield, 2016; Huang et al., 2023). At the subcortical level, hearing loss can also alter central auditory gain and excitatory-inhibitory balance (Ono and Ito, 2024). These changes may provide a sensory background for large-scale cortical reorganization.

This study was conducted exclusively in older individuals over the age of 60, who may possess distinct characteristics compared to younger populations. For instance, the total GEV was approximately 0.70. This indicates that part of the EEG variance was not explained by the seven-class model. This may reflect the heterogeneity of resting-state EEG in older adults (Voytek et al., 2015; Jabès et al., 2021; Kang et al., 2024). Importantly, the same pipeline and templates were used across groups. Thus, the modest GEV is unlikely to systematically bias group comparisons.

Several limitations should be noted. First, tinnitus status was not fully matched between groups, and tinnitus has been established to impact the EEG microstate. However, tinnitus was present in only a small number of participants (1/50 in the NH group; 8/45 in the ARHL group). In addition, tinnitus is common in ARHL and may form part of the clinical phenotype. Future studies with larger samples should examine hearing loss and tinnitus jointly. Second, none of the ARHL participants used hearing aids or actively sought amplification. Thus, this group mainly represented untreated mild-to-moderate ARHL. Hearing-related distress and motivation for amplification were not quantitatively assessed. These factors should be included in future work. Finally, the sample size of this study was moderate. However, because EEG signals in older adults tend to be noisier and exhibit greater variability, this may result in reduced sensitivity to detect group-level differences.

## 5 Conclusion

ARHL was associated with altered resting-state EEG microstate dynamics. These changes were mainly reflected in TPs. Individuals with ARHL showed reduced microstate D-to-G transitions and increased bidirectional C-to-F and F-to-C transitions. In addition, microstate G coverage and G-to-D transitions were lower in the ARHL group at the nominal significance level. The associations with PTA and SIN thresholds further suggest that these microstate alterations vary with the severity of hearing loss. Together, these findings indicate that chronic auditory decline may be linked to altered temporal organization of large-scale brain networks, involving attention-related, sensorimotor, posterior DMN-related, and anterior cingulo-frontal/insular dynamics. Resting-state EEG microstate analysis provides a comprehensive and systematic framework for studying brain network temporal organization induced by auditory decline.

## Data Availability

The original contributions presented in the study are included in the article/supplementary material, further inquiries can be directed to the corresponding authors.
